# The role of protein arginine deiminase 4-dependent neutrophil extracellular traps formation in ulcerative colitis

**DOI:** 10.3389/fimmu.2023.1144976

**Published:** 2023-04-18

**Authors:** Ping Wang, Dan Liu, Ziqi Zhou, Fangjun Liu, Yiming Shen, Qi You, Shiping Lu, Jie Wu

**Affiliations:** ^1^ School of Life Science and Technology, China Pharmaceutical University, Nanjing, China; ^2^ Department of Immunology and Microbiology, Tulane University, New Orleans, LA, United States

**Keywords:** neutrophil extracellular traps, peptidyl arginine deiminase 4, ulcerative colitis, intestinal inflammation, barrier function

## Abstract

**Background:**

Neutrophil extracellular traps (NETs) play an important role in the development and progression of ulcerative colitis (UC). Peptidyl arginine deiminase 4 (PAD4) is essential for the formation of NETs via catalyzing histone citrullination. This study mainly to explore the role of PAD4-mediated NETs in intestinal inflammation of dextran sulfate sodium (DSS)-induced UC.

**Methods:**

Acute and chronic colitis mouse models were established by supplementing DSS in drinking water. Colon tissues from colitis mice were analyzed for the level of PAD4 expression, citrullinated histone H3(Cit-H3), intestinal histopathology, and inflammatory cytokines secretion. Serum samples were tested for systemic neutrophil activation biomarkers. Colitis mice administered with Cl-amidine, a PAD4 inhibitor, and PAD4 knockout mice were investigated to detect NETs formation, intestinal inflammation, and barrier function.

**Result:**

We found the formation of NETs significantly increased in DSS-induced colitis mice and was correlated with disease markers. Blocking NETs formation by Cl-amidine or PAD4 genetic knockout could alleviate clinical colitis index, intestinal inflammation, and barrier dysfunction.

**Conclusion:**

This study provided a research basis for the role of PAD4-mediated NETs formation in the pathogenesis of UC and suggested that inhibition of PAD4 activity and the formation of NETs may be helpful for the prevention and treatment of UC.

## 1 Introduction

Epidemiological surveys have shown that the worldwide incidence of inflammatory bowel disease (IBD) has risen significantly in decades with an increased trend in younger ages (1). Ulcerative colitis (UC) is one of the major forms of IBD characterized by weight loss, abdominal pain, diarrhea, and bloody stools (2). The possible etiology of UC involves genetic, environmental, and immunological factors, as well as complex interactions between the gastrointestinal microbiota and the host organism (3).

Indeed, the hallmark of active UC is an abnormal immune response against luminal antigens. It is characterized by excessive immune cell infiltration, among which neutrophils are the first responder recruited to the site of inflammation where they recognize, engulf, and kill pathogens by producing reactive oxygen species (ROS) and antimicrobial peptides, releasing lytic enzymes and neutrophil extracellular traps (NETs) (4, 5). Previous studies showed that UC patients have significantly higher levels of NETs-related biomarkers than those in healthy controls (4, 6). And neutrophils isolated from UC patients are more likely to form NETs when stimulated *in vitro (* 4, 7, 8). Meanwhile, researchers have found remarkable increases in NETs and related proteins in mouse models of experimental colitis (4, 9, 10). Our lab has also shown that degradation of NETs via Staphylococcal nuclease (SNase) could recover intestinal barrier function and ameliorate colonic inflammation in mice (11, 12). However, how NETs formation contributes to mucosal inflammation in the colon has not yet been defined.

Histone citrullination is the prerequisite and trigger for NETs formation, which is mediated by peptidyl arginine deiminases (PAD) *via* converting arginines to citrullines (13–16). Peptidyl arginine deiminases 4 (PAD4) is the main PAD isoenzyme that is highly expressed in neutrophils under normal physiological conditions (17). The citrullination level of histone H3, chromatin decondensation and NETs-like structure formation are highly sensitive to the PAD4 activity (18). The use of Cl-amidine, a PADs inhibitor, in other autoimmune inflammatory disorders like systemic lupus erythematosus (SLE) and rheumatoid arthritis (RA) demonstrated that the level of NETs formation *in vivo* is closely correlated with site inflammation and pathogenesis (19, 20). Chumanevich et al. reported Cl-amidine alleviated colon inflammation manifestations in DSS-induced colitis (21). Our lab also proved that Cl-amidine reduced the clinical colitis index in 2,4,6-trinitrobenzene sulfonic acid-induced colitis mice (22). Yet the mechanism of action of PAD4 in colitis is still unclear.

In this study, we investigated how PAD4-mediated NETs control intestinal mucosal injury in DSS-induced acute and chronic colitis using PAD4 knockout (PAD4^-/-^) mice and Cl-amidine administered wide-type mice.

## 2 Methods and materials

### 2.1 Animals

Specific pathogen-free (SPF) male C57BL/6J mice (six to eight-week-old, weighing 20-25g) were purchased from Nanjing Qinglong Mountain Experimental Animal Breeding Center, Nanjing, China. The PAD4^-/-^ (KO) mice in C57BL/6J background and wild-type (WT) control mice (half male and half female, six to eight-week-old) were obtained from GemPharmatech Co., Ltd. All mice were acclimated and housed in the SPF-level Animal Experimental Center of China Pharmaceutical University (Nanjing, China). All animal experiments were approved by university animal ethics committees.

### 2.2 DSS-induced colitis mouse models

To establish an acute experimental colitis model, mice received 3% dextran sulfate sodium (DSS) (MP Biomedicals, USA) in drinking water for seven days (23). In the PAD4 inhibitor experiment, DSS-treated C57BL/6J mice were gavage fed by Cl-amidine (25mg/kg) daily, while the vehicle controls only received distilled water. Mice were all euthanized on Day 7.

To establish a chronic UC model, mice received two cycles of alternating 2.5% DSS (7 days) and distilled water washout period (7 days in the first cycle and 5 days in the second cycle) as previously described (24). Mice were euthanized on Day 22 for downstream analysis.

All animals had normal daily fluid intake. Body weight, stool consistency and hematochezia were monitored daily and used for disease activity index (DAI) calculation, which was scored as previously described (22).

### 2.3 Neutrophil isolation and NETs assay

Mouse spleen neutrophils were isolated using Percoll (Solarbio, Beijing, China) gradient (25) and NETosis was measured as previously published with modifications (26). In brief, fresh primary neutrophils were resuspended in RPMI-1640 (Biyuntian Biotechnology, Shanghai, China) supplemented with 10% FBS (ThermoFisher) and plated in 24-well plates at 10^6^ cells/well. After 1h incubation, cells were stimulated with ionomycin (10 μM, MedChemExpress, New Jersey, USA) for another 3h to form NETs. Cells were instantly stained with SYTOX green (1:1000, KeyGEN BioTech, Nanjing, China) for NETs quantification under dark. Images were acquired on an Axiovert 200M fluorescence microscope (Nikon, Shanghai, China).

### 2.4 Histopathological analysis

Mouse colon tissues were fixed in 4% paraformaldehyde (Servicebio, Wuhan, China) for 12 hours and embedded. Sections were processed and stained with hematoxylin and eosin (H&E) or Alcian blue (AB) for pathological evaluation. The histological score was evaluated according to the inflammatory infiltration and mucosal damage as described previously (27). Representative images were captured using a fluorescence inversion microscope (Nikon, Shanghai, China).

### 2.5 Immunohistochemistry and immunofluorescence

The colon paraffin sections were deparaffinized in xylenes and gradually rehydrated by sequential immersion in graded ethanol (absolute, 95%, 80% and 70% ethanol) (Nanjing Reagent, Nanjing, China) and finally in the water. Antigen retrieval was performed by microwave irradiation in citrate buffer. Slides were cooled down and washed with deionized water for further IHC or IF staining.

For IHC, colon sections were incubated with 3% H_2_O_2_ aqueous solution for 15 min to quench the endogenous peroxidase activity. After washing, tissue sections were incubated with Rabbit anti-Cit-H3 antibody (1:500 dilution, Abcam) overnight at 4°C. Slides then were washed three times and incubated with HRP-conjugated goat anti-rabbit antibody (1:200 dilution, Servicebio) for 30 min at room temperature. Finally, the sections were visualized with 3-3′-diaminobenzidine (DAB) and counterstained with hematoxylin.

For IF, activated colon sections were blocked with 10% bovine serum albumin (BSA, Sangon Biotech) for 30min at 37°C. Later, sections were stained with Ly6G (1:500; Servicebio), F4/80 (1:1000; Servicebio), CD11c (1:300; Servicebio), and CD3 (1:500; Servicebio). Photographs were taken on Pannoramic^®^ 250 FLASH slide scanner (Servicebio).

### 2.6 Myeloperoxidase enzyme activity

MPO is an abundant granule enzyme in neutrophils and MPO enzyme activity is used as an indicator for neutrophil infiltration (11). Colon tissues were homogenized in cold saline with a ratio of 1:10 (g/mL) and centrifuged at 12,000 rpm, 4°C for 15 min. The supernatant (10 μL) was added to a 96-well plate and reacted with 100uL of 3,3′,5,5′- Tetramethylbenzidine (TMB, Beyotime Biotechnology)) at 37°C for 5 min. The reaction was terminated with 2M H_2_SO_4_ and measured at 450 nm using a microplate reader (11).

### 2.7 ELISA

Supernatants were collected from tissue homogenates as mentioned in MPO enzyme activity section. The concentrations of IL-1β, IL-6, IL-17, TNF-α, MPO-DNA, and Cit-H3 were measured using commercial ELISA kits (Jiangsu Meimian Industrial Co., Ltd) per the manufacturer’s instructions.

### 2.8 Quantitative real-time PCR

After the mice were sacrificed, the colon pieces were placed in BioSample stabilizing Reagent (Accurate Biotechnology, Hunan, China), Total colon RNAs were isolated using RNAex Pro Reagent (Accurate Biotechnology, Hunan, China) according to the manufacturer’s instructions. cDNAs were synthesized using a cDNA Synthesis Super Mix kit (Accurate Biotechnology, Hunan, China). RT-qPCR analyses were performed to quantitate the relative mRNA expression using SYBR Green qPCR Master Mix kit (Accurate Biotechnology, Hunan, China) with primers listed in **Supplementary Table**. GAPDH was used as the endogenous reference, and gene expressions were normalized to GAPDH using the 2^-ΔΔCT^ algorithm (28).

### 2.9 Western blotting

Colon tissue was snap-frozen and homogenized in RIPA buffer (Sangon Biotech, Shanghai, China) containing protease inhibitors on ice. The homogenate was centrifuged at 12,000 rpm for 10 min at 4°C and the total protein was quantified using a BCA (Beyotime Biotechnology, Shanghai, China) assay. Equal amounts of proteins were mixed with loading buffer, loaded on gradient gels (12% SDS-PAGE), and then transferred to a PVDF membrane. The membrane was blocked with 5% milk in TBST and incubated with primary antibodies at 4°C overnight as listed: rabbit polyclonal anti-Cit-H3 (1:1000, Abcam); rabbit polyclonal anti-GAPDH (1:2000, Servicebio); rabbit polyclonal anti-PAD4 (1:5000, Proteintech); rabbit polyclonal anti-MPO (1:3000, Proteintech); rabbit polyclonal anti-occludin (1:3000, Proteintech). After washing, the blot was finally incubated with HRP-conjugated goat anti-rabbit secondary antibody (1:5000, Proteintech) for 1h at room temperature. Bands were visualized using a chemiluminescent substrate (Sangon Biotech) and were quantified based on the endogenous GAPDH level using ImageJ software.

### 2.10 Flow cytometry staining

Single-cell suspensions from mesenteric lymph nodes (MLNs) were isolated and stained for immune cell phenotyping as previously described (29). Briefly, single cells were harvested in FACS buffer, fixed, and incubated with FITC-anti-mouse CD4 (1:50, clone RM4-5, BD Biosciences) for 40min at 4°C. After washing, cells were permeabilized with Cytofix/Cytoperm (BD Biosciences) and stained with intracellular biomarkers for 40min at 4°C. Antibodies PE-cy7-anti-mouse RORγt (1:50, clone B2D; BioLegend) and PerCP-anti-mouse Foxp3 (1:50, clone MF23; BioLegend) were used to analyze the proportion of Th17 and Treg cells respectively. Positive cells were measured by a BD Accuri C6 flow cytometer and analyzed by FlowJo software.

### 2.11 Statistical analysis

All quantitative results were expressed as mean ± standard error of the mean (SEM). Statistical analyses were performed using GraphPad Prism version 8.0. Differences between groups were compared using unpaired Student’s *t*-test or one-way ANOVA test. Correlation analysis was performed by the Pearson correlation coefficient method. P < 0.05 were considered statistically significant.

## 3 Results

### 3.1 PAD4 inhibitor diminished NETs formation and ameliorated DSS-induced acute colitis

**Figure 1:**
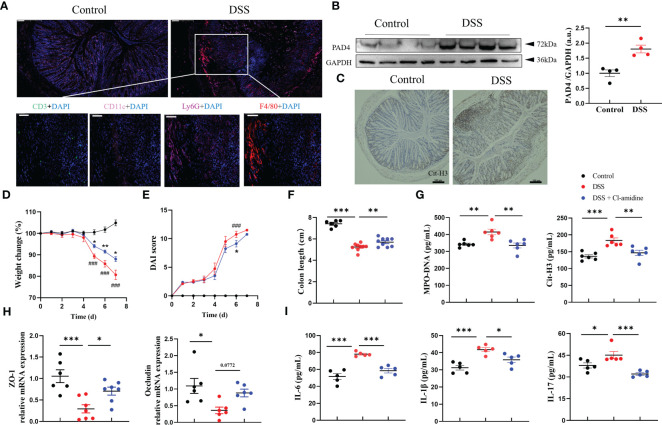
PAD4 Inhibitor diminished NETs formation and ameliorated DSS-induced acute colitis. **(A)** Representative multicolor immunofluorescence staining of colon sections, stained for CD3 (green), CD11c (pink), Ly6G (rose-carmine), F4/80 (red) and DNA (DAPI, blue). **(B)** Western blot analysis of PAD4 in colon tissue. **(C)** Representative immunohistochemical images of Cit-H3 positive cells in colon sections. The effect of Cl-amidine on disease indexes in colitis mice: Body weight **(D)**, DAI score **(E)** (^###^ *P<0.001* compared with Control group, *P<0.05, **P<0.01 compared with DSS group), colon length **(F)** (n=8-10). **(G)** The levels of MPO-DNA and Cit-H3 in serum (n=6). **(H)** RT-qPCR of ZO-1 and Occludin in colon tissue (n=6-8). **(I)** The levels of IL-6, IL-1β and IL-17 in colon tissue (n=5). Bars represent 100 μm. *P<0.05, **P<0.01, ***P<0.001.

First, we established an experimental mouse model of acute colitis using DSS to evaluate the NETs activity. This DSS-induced colitis model is characterized by significantly more weight loss, higher disease activity and shorter colon length than the naïve littermates (**Supplementary Figures S1A–C**). More importantly, we found noticeable inflammation in the colon with the accumulation of Ly6G+ neutrophils and F4/80+ macrophages whereas the minimum of CD3+ lymphocytes and CD11c+ dendritic cells were presented (**Figure 1A**). As PAD4 and Cit-H3 are closely associated with NETs formation and subsequent colitis (4), we also tested their expressions in our model. The expression of PAD4 protein was significantly enhanced in the inflamed colon compared with normal tissue (**Figure 1B**). Similarly, the Cit-H3 positive cells were dramatically increased in the DSS-treated samples (**Figure 1C**). We also verified that the weight change and colon length in colitis mice were negatively correlated with Cit-H3 expression and MPO-DNA level [MPO forms complexes with DNA in serum (30)] (**Supplementary Figure 1D**). Therefore, we confirmed the acute colitis model establishment which features neutrophil hyperactivation and inflammation.

Next, we determined whether PAD4-associated NETs release contributed to colitis. DSS-treated mice were given Cl-amidine, a PAD4 inhibitor (31). We observed that Cl-amidine treatment reduced the severity of DSS-induced colitis with less weight loss, lower DAI score, and longer colon length (**Figures 1D–F**). Again, the NETosis-associated indicators, serum MPO-DNA and Cit-H3 were significantly increased in DSS-treated mice while decreased after Cl-amidine treatment (**Figure 1G**). Gut barrier integrity maintained by tight junction proteins like ZO-1 and occludin is another sign of colitis severity (32). We found that the mRNA levels of ZO-1 and occludin were significantly decreased in DSS-induced colitis samples but dramatically increased after Cl-amidine treatment similar to the levels in naïve controls (**Figure 1H**). Tissue inflammation biomarkers like classical colitis cytokines were also examined. The upregulation of proinflammatory cytokine production, including IL-6, IL-1β and IL-17, induced by DSS treatment was greatly alleviated by this PAD4 inhibitor Cl-amidine (**Figure 1I**; **Supplementary Figure S2**). Taken together, these data demonstrated that PAD4-related NETosis is critical for colitis formation.

### 3.2 PAD4 deficiency relieved colitis severity potentially due to reduced NETs formation

**Figure 2:**
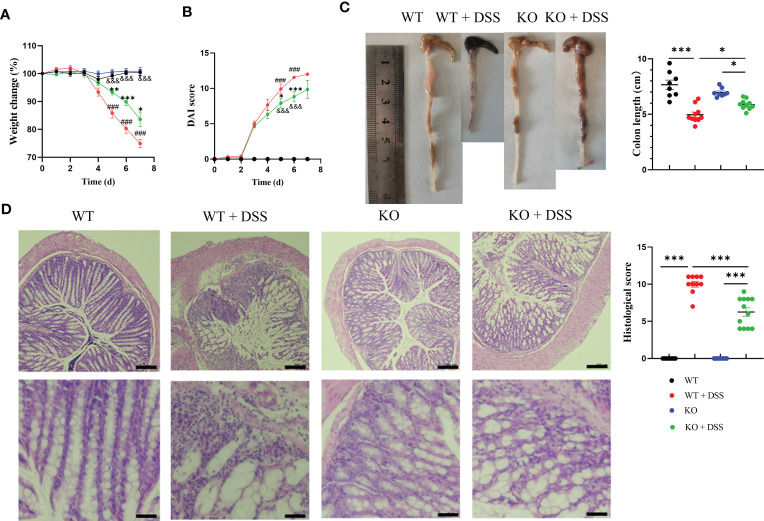
The effect of PAD4 deficiency on disease indexes in acute DSS-induced colitis mice. **(A)** Weight change. **(B)** DAI score (^###^ *P<0.001* compared with WT group; *P<0.05, ***P<0.001 compared with WT+DSS group; ^&&&^ *P<0.001* compared with KO group). **(C)** Colon length (n=8-10). **(D)** Microscopic total damage scores and representative H&E-stained colon sections of mice (n=10-12). Bars represent 100 µm in the low magnification (10×; the upper panel) and 50 µm in the higher magnification (20×; the bottom panel) images. The histological scores were assessed as described previously (27). *P<0.05, **P<0.01, ***P<0.001.

To further characterize the functional contribution of PAD4 in colitis, we utilized PAD4 global knockout (PAD4^-/-^) mice in C57BL/6J background. Colitis severity was dramatically decreased in PAD4^-/-^ mice after DSS treatment, which displayed a smaller weight loss (**Figure 2A**), lower DAI score (**Figure 2B**) and minimal lethality (**Supplementary Figure S3**), compared with their wild-type controls. The aberrant colon phenotypes were also attenuated in the absence of PAD4. Colon lengths in the DSS-treated PAD4^-/-^ mice were recovered dramatically, though not in full, compared with DSS-treated wildtypes (**Figure 2C**). Neutrophil infiltration in the colon was significantly reduced in the PAD4^-/-^ mice than the wild-types after DSS induction shown by H&E staining, histological score (**Figure 2D**) and MPO activity (**Supplementary Figure S3**).

**Figure 3:**
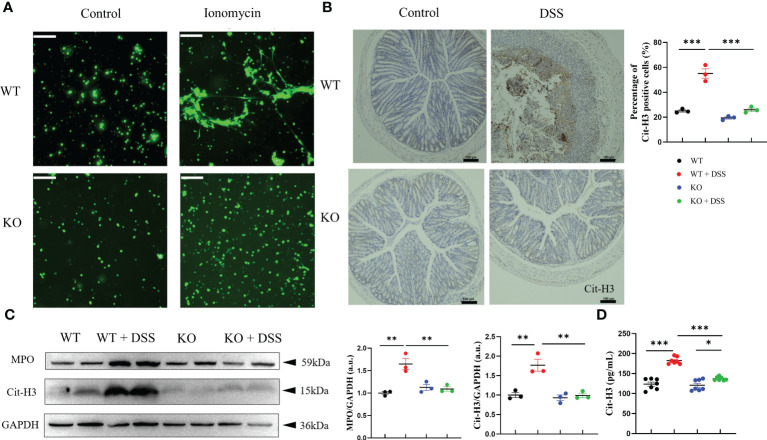
PAD4 deficiency diminished NETs formation *in vivo* and *in vitro*. **(A)** NETs formation ability in neutrophils isolated from WT mice and KO mice. **(B)** Representative immunohistochemical images of Cit-H3 positive cells in colon sections (n=3). **(C)** Western blot analysis of MPO and Cit-H3 in colon tissue (n=3). **(D)** The concentration of Cit-H3 in serum (n=7). Bars represent 100μm. *P<0.05, **P<0.01, ***P<0.001.

Furthermore, we demonstrated whether the milder colitis in DSS-treated PAD4^-/-^ mice was caused by NETs regulation. Primary neutrophils were stimulated with ionomycin and stained for NETs quantification, which showed that neutrophils isolated from PAD4^-/-^ mice formed significantly less DNA extrusion (also known as NETs formation) than their wild-type counterparts (**Figure 3A**). The reduction in NETs formation was also observed *in vivo*. IHC staining showed that the Cit-H3 positive cells in colon sections were statistically reduced (**Figure 3B**). Consistently, the expression of MPO and Cit-H3 were significantly decreased both in PAD4^-/-^ mice colons shown by western blotting and serum shown by ELISA (**Figures 3C, D** **)**. Together, the PAD4 deficiency diminished NETs formation *in vivo* and *in vitro* and undoubtedly contributed to the milder DSS-induced acute colitis.

### 3.3 PAD4 knockout improved gut barrier dysfunction and suppressed inflammatory responses in acute DSS-induced colitis

**Figure 4:**
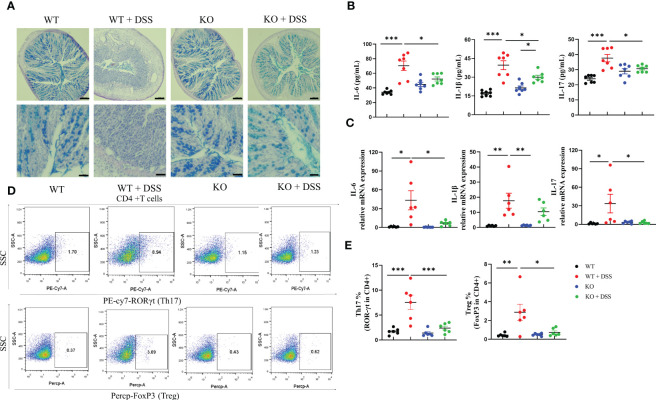
PAD4 deficiency improved gut barrier dysfunction and suppressed inflammatory responses in acute DSS-induced colitis. **(A)** Representative AB staining images of colon tissue. Bars represent 100 µm (10×; the upper panel.) and 50 µm (20×; the bottom panel) images. **(B)** The protein expression of IL-6, IL-1β and IL-17 in colon tissues (n=6-8). **(C)** RT-qPCR results of cytokines IL-6, IL-1β and IL-17 in colon tissues (n=6-8). **(D)** Representative flow cytometry plots of Th17 cells and Treg cells in MLNs. **(E)** Quantitative analyses of the percentages of Th17 and Treg cells in MLNs (n=6). *P<0.05, **P<0.01, ***P<0.001.

Goblet cells have a well-appreciated role in maintaining barrier integrity via mucus secretion, whose loss of function is a hallmark of IBD. Alcian Blue (AB)- stained colon tissue sections showed that goblet cells were rare and more condensed in DSS-induced colitis mice but restored in induced PAD4^-/-^ mice (**Figure 4A**). NETs formation is well-documented about the immune-modulatory functions in disease which facilitate inflammatory cascades (33). Therefore, we measured the proinflammatory cytokine production in colon tissues that DSS-induced acute colitis is accompanied by increased cytokine expression shown by ELISA and qRT-PCR, including IL-6, IL-1β and IL-17. Interestingly, the overexpression in cytokines was greatly countered under the depletion of PAD4 (**Figures 4B, C** **)**, suggesting the importance of PAD4 in colon inflammatory responses. In addition, the pathogenesis of IBD usually involves the lymphatic system, especially the mesenteric lymph nodes (MLNs) where the immune responses are linked to inflammation pathophysiology (34). Our results showed DSS-induced acute colitis is marked by the expansion of Th17 and Treg cells in the MLNs of wild-type mice. Notably, depletion of PAD4 could redirect the impaired immune responses to the normal status (**Figures 4D, E** **)**.

### 3.4 PAD4-mediated NETosis also contributed to chronic colitis in similar mechanisms

**Figure 5:**
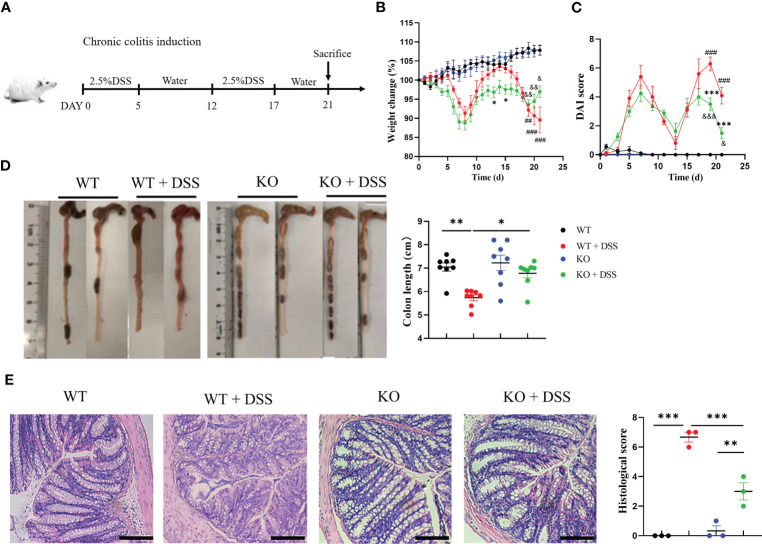
PAD4 deficiency also improved disease indexes in chronic DSS-induced colitis. **(A)** Two cycles administration of DSS induced chronic colitis. **(B)** Weight change. **(C)** DAI score, (^###^ *P<0.001*, ^##^ *P<0.001* compared with WT group; *P<0.05, ***P<0.001 compared with WT+DSS group; ^&^ *P<0.05*, ^&&^ *P<0.01*, ^&&&^ *P<0.001* compared with KO group). **(D)** Colon length (n=6-8). **(E)** Microscopic total damage scores and representative H&E-stained colon sections of mice (n=3). Bars represent 50 μm. *P<0.05, **P<0.01, ***P<0.001.

To fully characterize the contribution of PAD4 and associated NETosis in UC, we also established a chronic colitis mouse model induced by two cycles of 2.5% DSS (**Figure 5A**). Similar to the acute colitis model, the mouse weight loss and DAI score were increased when treated with 2.5% DSS but recovered partially in the PAD4^-/-^ mice (**Figures 5B, C** **)**. In the end, the colon length was measured which was similar to the normal length in DSS-treated PAD4^-/-^ mice (**Figure 5D**). The typical colitis lesions, such as crypt abscess and absence of goblet cells shown in DSS-induced wild-types, were dramatically rescued in the PAD4^-/-^ counterpart (**Figure 5E**).

**Figure 6:**
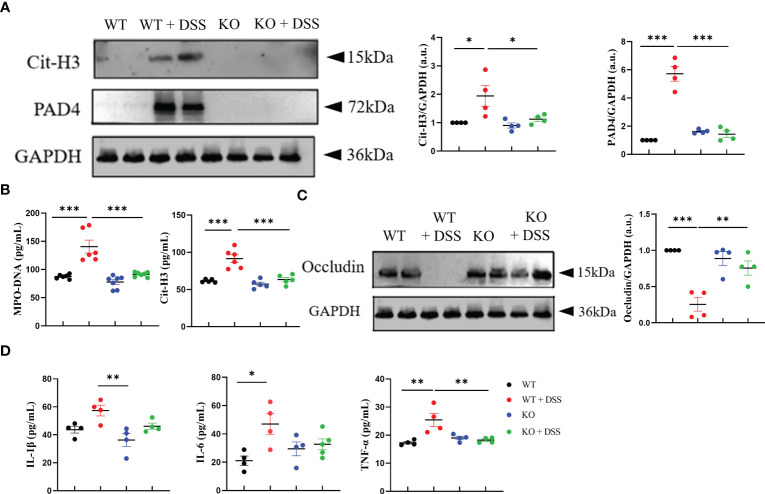
PAD4 deficiency improved intestinal integrity and alleviated intestinal inflammation in chronic colitis mice. **(A)** Western blot analysis of Cit-H3 and PAD4 in colon tissue (n=4). **(B)** The level of MPO-DNA and Cit-H3 in serum (n=6). **(C)** Western blot analysis of Occludin in colon tissue (n=4). **(D)** The levels of IL-1β, IL-6 and TNF-α in colon tissue (n=6). *P<0.05, **P<0.01, ***P<0.001.

Moreover, we investigated the connection between the inhibition of chronic colitis and NETs activity in absence of PAD4. First, western blotting results showed a significant decrease of Cit-H3 expression in PAD4^-/-^ colons than the controls after DSS induction (**Figure 6A**). Consistently, the abnormal overexpression of serum MPO-DNA and Cit-H3 caused by DSS induction was effectively reversed in PAD4^-/-^ mice (**Figure 6B**). Indeed, those two NETs activity biomarkers were closely correlated with chronic colitis pathology (**Supplementary Figure S4**).

To further explore how PAD4 deficiency impacts colon tissues in this chronic inflammation setting, we evaluated intestinal integrity and proinflammatory cytokine production. The decrease of occludin protein caused by DSS treatment was rescued in PAD4^-/-^ mice (**Figure 6C**). Also, the IBD-related proinflammatory cytokines, such as IL-1β, IL-6 and TNF-α (35), were markedly reduced in DSS-treated PAD4^-/-^ colon tissue, as compared to the controls (**Figure 6D**). These data suggested that PAD4 likely contributes to the pathogenesis of chronic colitis in mechanisms similar to those in acute colitis.

## 4 Discussion

This study aimed to investigate the role of PAD4-mediated NETs in intestinal inflammation of DSS-induced UC. Consistent with previous studies (4, 21), our data indicated that the levels of MPO-DNA, Cit-H3 in serum and PAD4 in colonic tissues of UC mice were increased after DSS induction. And the levels of NETs indicators were positively related to the disease severity. PADs especially PAD4 plays an important role in mediating histone H3 citrullination, which disrupts ion interactions like hydrogen bonds in chromatin and leads to chromosomal disintegration. This step triggers the subsequent formation of NETs (9), providing a basis to further study the pathogenetic role of PAD4-based NETs in UC.

Studies in other systems have shown that various modalities such as PADs inhibitors (21, 36, 37), DNA-degrading DNases (4), and SNase (11) can reduce the level of NETs *in vivo*. Cl-amidine is an effective, small molecular inhibitor of PADs. In our study, it showed that Cl-amidine alleviates colitis symptoms including reduced weight loss and DAI scores, and increased colon length. Moreover, Cl-amidine increased the expression of tight junction proteins like ZO-1 and occludin in colon tissues. Furthermore, the results proved that Cl-amidine decreased the expression of pro-inflammatory cytokines and NETs-related proteins. These evidences indicated that inhibition of PADs activity and the formation of NETs can ameliorate the DSS-induced colitis.

Accumulating evidence suggests that PAD4 and its polymorphisms-mediated histone citrullination are crucial in NETosis induced by various physiological stimuli, such as microorganisms and crystals (33). Among the PADs family, only PAD4 possesses a canonical nuclear localization signal and has been more extensively studied for its role in autoimmune diseases (38, 39). Therefore, we established DSS-induced acute and chronic colitis models based on PAD4 deficient mice. In line with data from previous studies (3, 11), we found intestinal inflammation and abnormal intestinal barrier function in colitis mice. Bacterial/pathogenic product entry such as LPS (lipopolysaccharides) has been proven to be the triggering factor for the activation of PAD4 and NETs formation (33). Our previous study has demonstrated that circulating LPS concentration in mice increased with DSS induction (11). Consistently, the levels of NETs indicators were significantly increased in two animal models. Depletion of PAD4 significantly reduced NETs formation *in vitro* and *in vivo* under stimulations. More importantly, PAD4 deficiency improved the disease manifestations of induced acute and chronic colitis. These improvements involve lower disease activities, better colon barrier function and less inflammation infiltration, which all re-emphasize the tight bonds between NETs formation and UC pathogenesis.

Our findings also supported the possibility to regain immune balance via controlling PAD4 activity. As reported previously, DNA and cellular contents released by NETs can effectively induce the polarization of naïve T cells to Th1 and Th17 cells (40, 41). Other researchers found that NETs promote the differentiation of naïve CD4+ T cells into Treg cells (42). Our results showed the increase of Th17 and Treg cells in the MLNs of DSS-induced acute colitis mice which both reversed after silencing PAD4, consisting with the change in the level of NETs. We speculated that Treg cells are regulating rather than pathogenic in UC. Although Tregs cell are increasing in response to gut disease inflammation, they may not be at a high enough number to compensate the defects (43). However, further works are needed to better answer this question. IL-17, a proinflammatory cytokine predominantly secreted by Th17 cells, was concluded to promote tissue inflammation via recruiting myeloid cells and promoting proinflammatory mediators (44). In our study, we observed that DSS-induced colitis was characterized by excessive inflammatory indicators, such as the upregulation of proinflammatory cytokines (IL-17, IL-6, IL-1β) in the colon and prevailing Th17/Treg cells in the MLNs. Instead, the aberrant inflammation responses were retuned, at least partially, with much lower aforementioned proinflammatory mediators in DSS-treated PAD4^-/-^ mice, clearly indicating that PAD4 is strongly associated with intestinal inflammation and abnormality of immune system.

**Figure 7:**
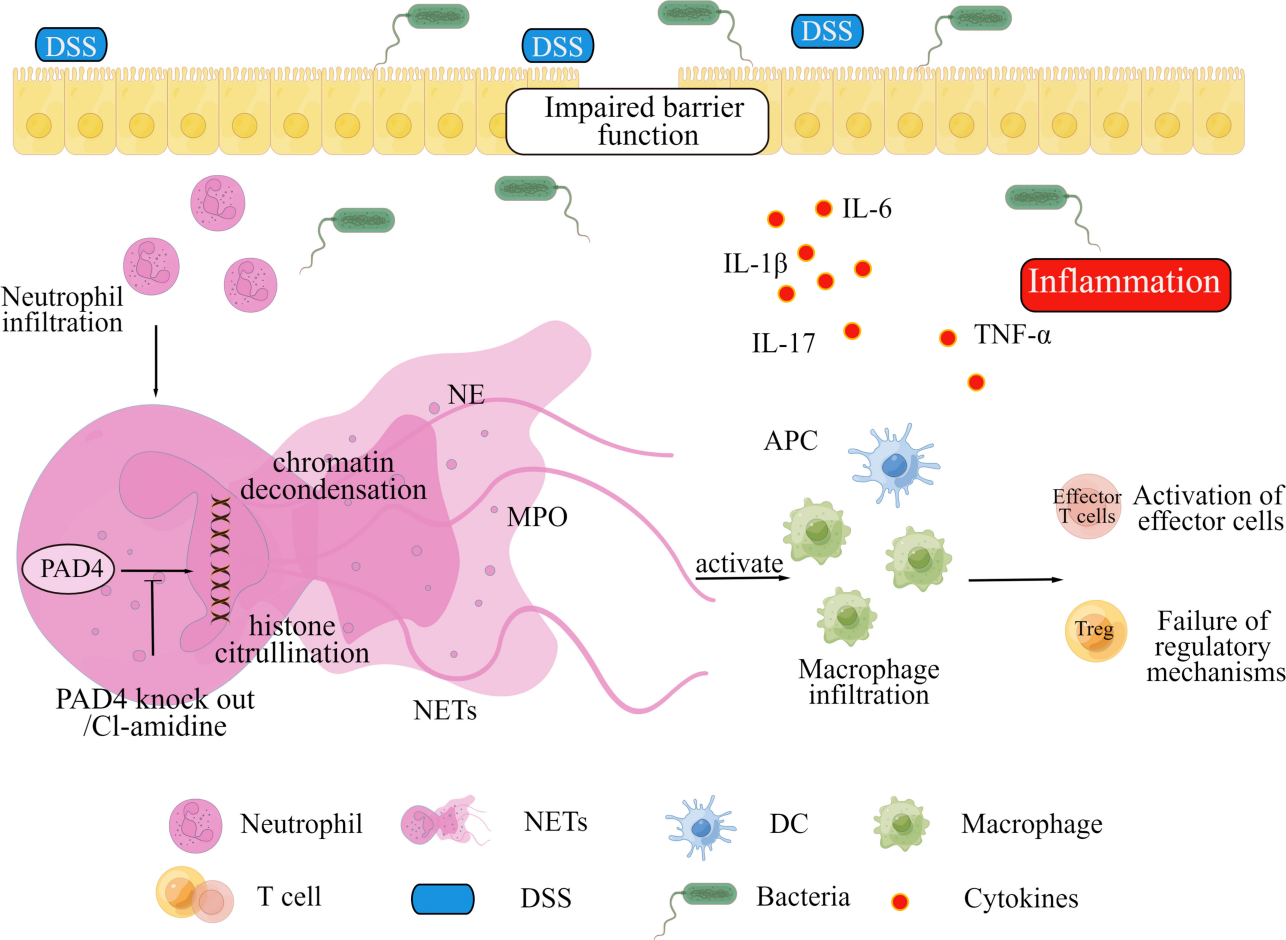
A proposed mechanism for PAD4-mediated NETs to mediate intestinal mucosal injury in DSS-induced colitis.

**Figure 7** showed a schematic diagram to explain the role of PAD4-mediated NETs in intestinal inflammation and mucosal injury. The mouse intestinal barrier function is significantly disrupted by DSS treatment. Subsequent intestinal epithelial cells apoptosis and microbial invasion induce abnormal NETs formation, which triggers downstream inflammation responses and forms colitis eventually. Blocking NETs formation by PAD4 inhibitor or PAD4 genetic knockout could alleviate barrier dysfunction and intestinal inflammations in colon, and ameliorate local colitis. However, further explorations on the specific molecular mechanisms are needed.

In conclusion, this study proves that PAD4-mediated NETs formation is indispensable for UC pathogenesis, which greatly contributes to intestinal inflammation, abnormal intestinal barrier function, and immune imbalance. Therefore, blocking NETs formation using PAD4 inhibitor like Cl-amidine is a promising therapeutic approach for the prevention and treatment of UC.

## Data availability statement

The original contributions presented in the study are included in the article/**Supplementary Material**. Further inquiries can be directed to the corresponding authors.

## Ethics statement

The animal study was reviewed and approved by Animal ethics committees in China Pharmaceutical University.

## Author contributions

PW, DL, ZZ, FL, YS, and QY designed and performed experiments. DL and PW analyzed experimental data and drafted the manuscript. JW and SL designed the study, supervised the project and drafted the paper. All authors contributed to the article and approved the submitted version.
