# Supplementary material for: The role of protein arginine deiminase 4-dependent neutrophil extracellular traps formation in ulcerative colitis
Source: Front Immunol. 2023 Apr 18;14:1144976. doi: 10.3389/fimmu.2023.1144976 (PMC10151647; doi:10.3389/fimmu.2023.1144976)

Supplementary Material

**The Role of Protein Arginine Deiminase 4-Dependent Neutrophil Extracellular Traps Formation in Ulcerative Colitis**

Ping Wang^a,1^, Dan Liu^a,1^, Ziqi Zhou^a^, Fangjun Liu^a^, Yiming Shen^a^, Qi You^a^, Shiping Lu^b,*^, Jie Wu^a,*^

*******Corresponding authors:**

Professor Jie Wu, School of Life Science and Technology, China Pharmaceutical University, Nanjing 211198, China. Email: [wujie@cpu.edu.cn](mailto:wujie@cpu.edu.cn)

Dr. Shiping Lu, Department of Immunology and Microbiology, Tulane University, New Orleans, United States. Email: [slu5@tulane.edu](mailto:slu5@tulane.edu)

# Supplementary Figures and Table

## Supplementary Figures


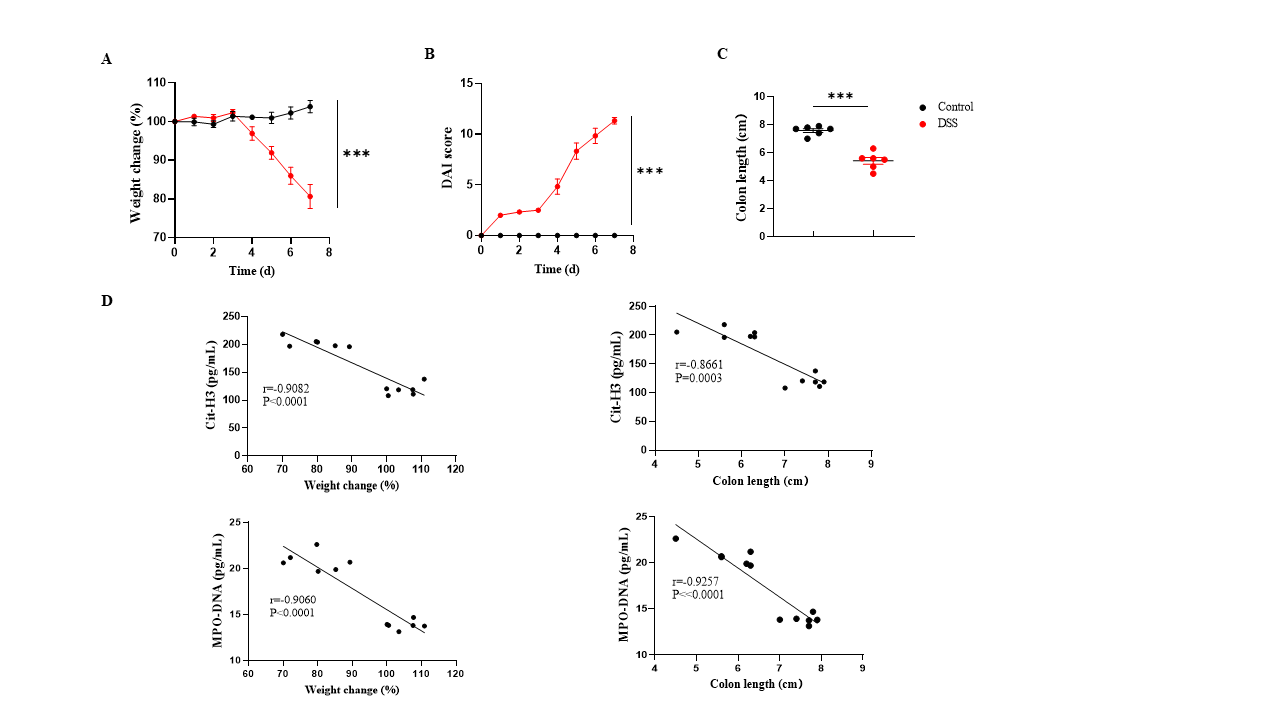


**Supplementary Figure 1.** Acute Colitis Constructed and Correlation between NETs formation and Acute Colitis. Weight change (**A**), DAI score (**B**), and colon length (**C**) (n=6). (**D**) The level of Cit-H3 and MPO-DNA in the serum correlated with body weight loss and colon length (n=12). ****P <0.001*.

**
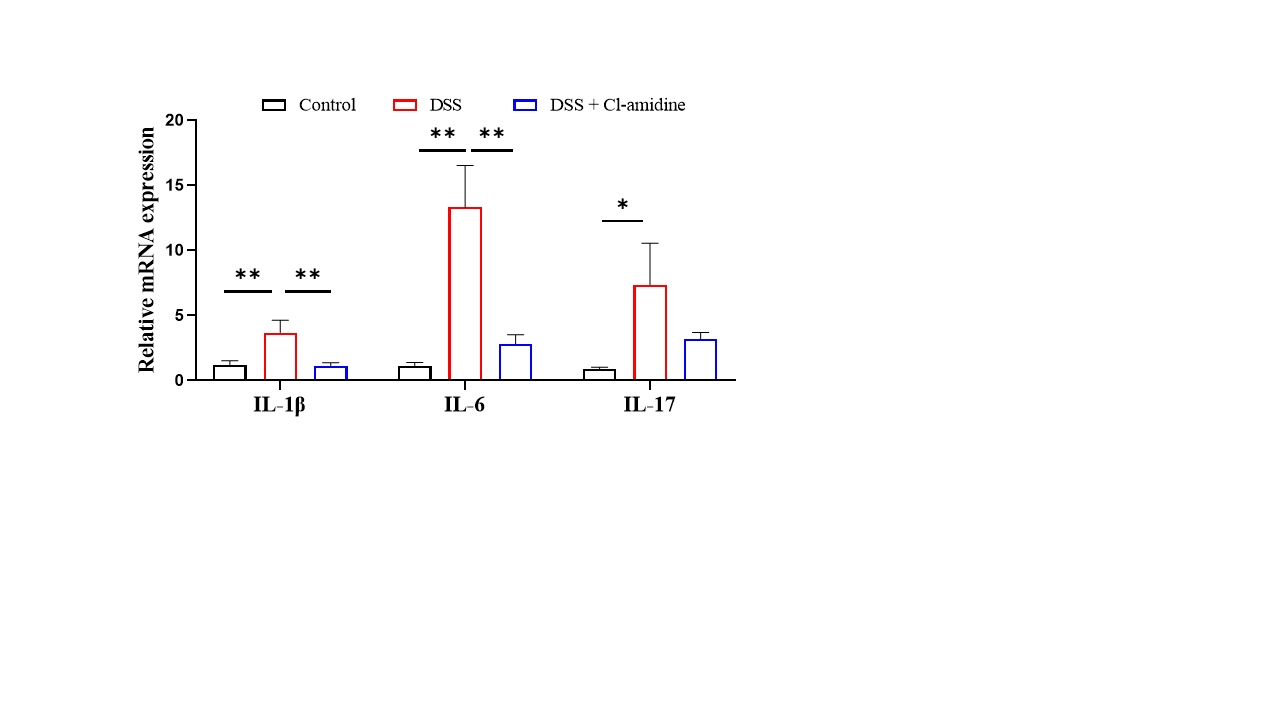
**

**Supplementary Figure 2.** PAD4 inhibitor relieved proinflammatory cytokine production in acute DSS-induced colitis. RT-qPCR of IL-6, IL-1β and IL-17 in colon tissue (n=5-6). **P<0.05, **P<0.01*.

**
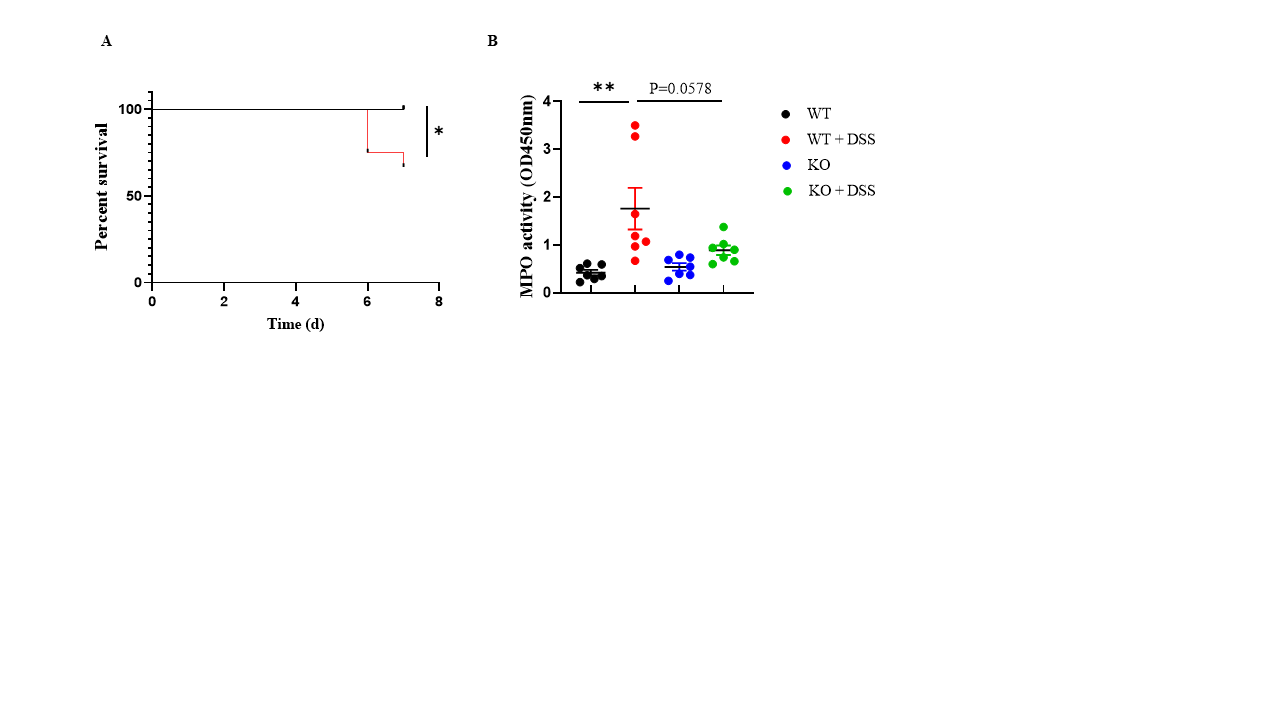
**

**Supplementary Figure 3.** PAD4 deficiency also improved disease indexes in chronic DSS-induced colitis. (**A**) Percent survival of mice in each group (n=8-12). (**B**) MPO activity in colon tissue (n=7). **P<0.05,* *** P<0.01*.

**
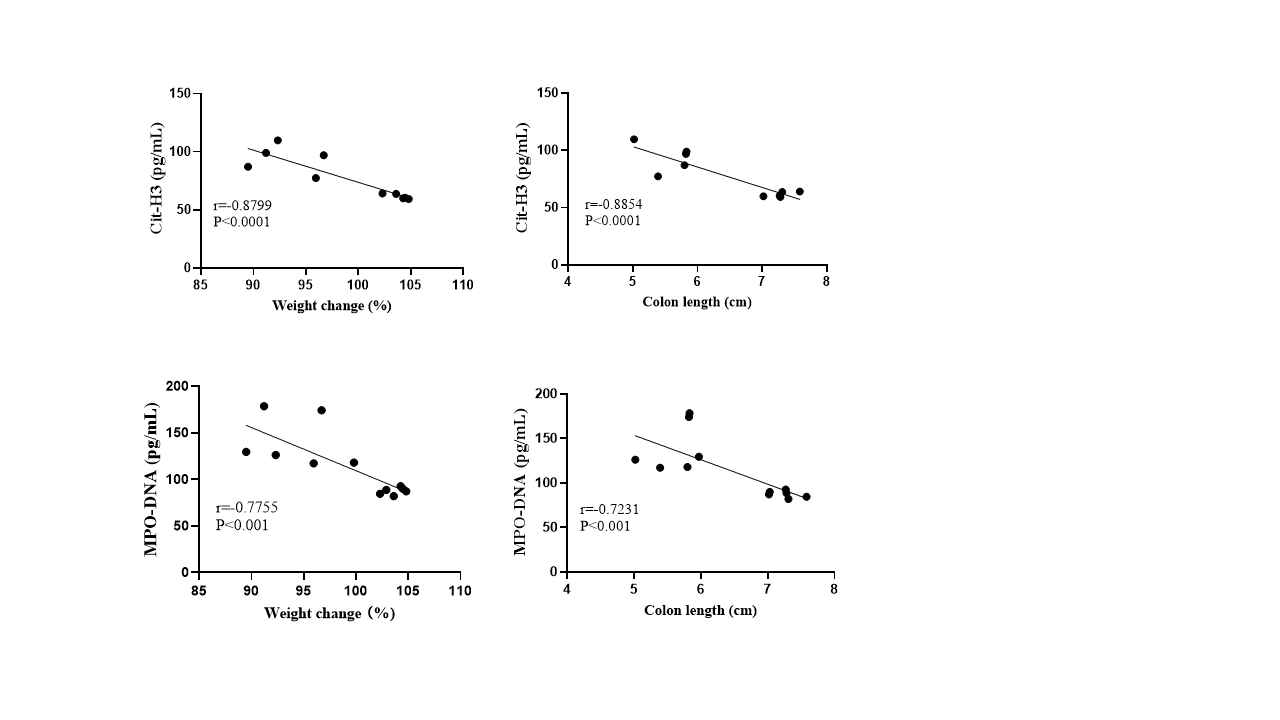
**

**Supplementary Figure 4.** Correlation between NETs formation and chronic colitis. The level of Cit-H3 and MPO-DNA in the serum of chronic colitis mice correlated with body weight loss and colon length (n=12).

## Supplementary Table

Primer sequence


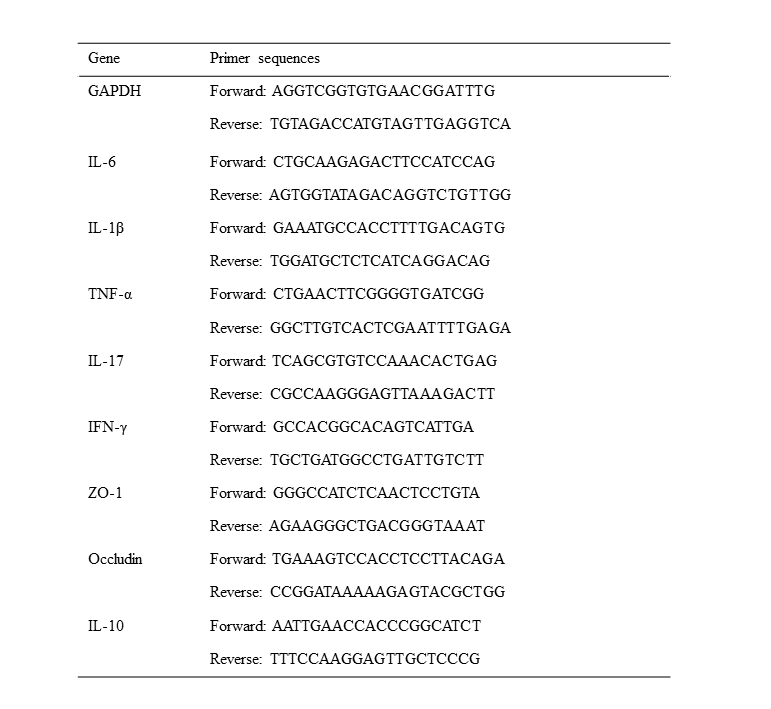

Supplement: Supplementary file 1 [file DataSheet_1.docx]
